# Accessibility to health services among migrant workers in the Northeast of Thailand

**DOI:** 10.12688/f1000research.11651.1

**Published:** 2017-06-22

**Authors:** Suprawee Khongthanachayopit, Wongsa Laohasiriwong

**Affiliations:** 1 Faculty of Public Health, Khon Kaen University, Khon Kaen, 40002, Thailand; 2 Research and Training Center for Enhancing Quality of Life of Working-Age People, Faculty of Public Health, Khon Kaen University, Khon Kaen, 40002, Thailand

**Keywords:** accessibility, health service, migrant workers, curative

## Abstract

**Background**. There is an increasing trend of trans-border migration from neighboring countries to Thailand. According to human rights laws, everyone must have access to health services, even if they are from other nationalities.  However, a small minority of health personnel in Thailand discriminate against immigrant workers, as they are from a lower financial bracket.

**Methods**. This cross-sectional study aims to determine the prevalence of accessibility to health services and factors associated with access to health services among migrant workers who work along the Northeast border of Thailand. A total of 621 legal migrant workers were randomly selected to respond to a structured questionnaire about the satisfaction of health services, using the 5As of health services: availability; accessibility; accommodation; affordability; acceptability.  Associations between independent variables and access to health services were analysed   using multiple logistic regression analysis.

**Results**. The results indicated that the majority of these registered migrant workers were female (63.9%) with an average age of 29± 8.61 years old, and were married (54.3%). Most of the workers worked at restaurants (80%), whereas only 20% were in agricultural sectors. Only 14% (95% CI: 11-17%) of migrant workers had access to health services. The factors that were significantly associated with accessibility to health service experienced ill health during the past one year (OR = 2.48; 95%CI; 1.54–3.97; p-value<0.001) **;** have been married (OR = 2.32; 95% CI: 1.40 – 3.90; p-value <0.001).

**Conclusions**. Most of the migrant workers could not access health services. The ones who did access health services were married or ill.

## Introduction

Mobilization of people across borders is widely spread around the world. There has been an increasing trend of migrant workers in Thailand, who are allowed to work all over the country. These individuals have increased by 13.18% since 2013, to comprise 87.99% of workers in 2014, totaling over 3 million individuals. These migrant workers are mostly from three nationalities: Burmese, Laotian and Cambodian ^1^. The workers’ physical appearance, language, and culture are quite similar to the Thai population, which causes the numbers of migrant workers and patients from neighbouring countries to increase annually ^2^. The country is in need of migrant workers for jobs that are mostly labour intensive both in agricultural and industrial sectors, which can be of a risky nature with lower wages. The number of migrants from many countries has rapidly increased as a result of economic development activities, trade and tourism along Thai borders. The growth of immigration is clearly seen, especially in the special economic zones, and Thailand is also an ASEAN Member State since December 2015.

The migrant workers mainly work as unskilled labour in dirty, dangerous and degrading conditions that leaves them exposed to a higher risk of communicable diseases, such as tuberculosis ^3^. From the literature it is noted that 40% of migrant workers do not have a health insurance card, which results in lower access to healthcare services compared to those with a health insurance card ^4^. It is mandatory that government healthcare services in the border provinces should serve these foreign patients, whether they can afford the medical expense or not. Several government healthcare institutes have used the budget allocated for Thai patients to support foreign patients ^5^. However, in 2015 the Thai government attempted to solve these problems by allowing foreigners and migrant workers to purchase a health insurance card with different coverage periods and extended the coverage to the foreign workers. Even a migrant worker who is legally registered with the Ministry of Labour has numerous difficulties in using a government health insurance card, for example the employer confiscates the health card from the workers, or the workers prefer private clinics due to inadequate attention in public hospitals ^6^. This obstructs migrant workers from having access to good healthcare. In addition, there are other factors, such as communications barriers, frustrations in contacting the government officers at the hospital, the distance from their residential areas or work place to the public hospital, that have hindered their access to health services, which, according to human rights, migrants must have equity of access to health care.

The concept of accessibility is a central objective of many health care systems. Nevertheless, there are substantial challenges to achieving this goal of health security for migrants. Access and how they experience their access to health service is important for the policy maker. A literature review of studies on accessibility to health services of migrant workers are limited, especially in Thailand. Data on accessibility to health service are not consistent and there are not enough studies on the given factors ^7^. The literature on health and access to care of migrants is limited and different in focus and quality ^8^. A previous study found that the migrant workers experienced alienation and inequality when they were treated at healthcare services ^9^. Therefore, there is still ambiguity in the knowledge regarding the current situation of migrant workers in the Northeast and associated factors during their work in Thailand. This study examines the factors associated with access to health services among legal migrant workers in the Northeast of Thailand.

## Methods

This cross-sectional study aims to examine the prevalence of accessibility to health services and factors associated with access for legal migrant workers in the Northeast of Thailand. The study applied the concept of access developed by Penschansky & Thomas in 1981 ^10^. The accessibility to health services in this study focused on satisfactory health services in terms of availability, accessibility, accommodation, affordability and acceptability (the 5As). To avoid recall bias, we trained the interviewer and carefully asked the questions in the migrants’ language (LAO).

### Study participants

The inclusion criteria were legal migrant workers, who were not of Thai nationality, but from LAO, and had registered as migrant workers with the Department of Employment, the Ministry of Labor, and had been working in Nakhon Phanom, Mukdahan and NongKhai province. The participants were migrant workers who had stayed in Thailand and had expired work permits dated 31 March 2016. Migrants were then selected randomly from a list once they re-registered.

The required sample size was estimated by using a formula for multiple logistic regression. ^11^, to identify relationships between multiple independent variables and a dichotomous dependent variable. Hence, the sample size was 547, with 15% increase to allow for potential non-responders. Therefore, the total number of samples was 629 individuals. Due to incompletion of some questionnaires only 621 samples were included in this study.

The participants were selected in this study by systemic random sampling from the name list of re-registered migrant workers from three provinces that were located in the North east part of Thailand.

### Accessibility questionnaire

When investigating access, we classified the dichotomous dependent variable into two groups: access and non - access. The questionnaire tool was developed from reviewing literature ^10, 12, 13^ and was also pretested among 30 workers in Loei province, which is a different area from the data collection site. Most of these workers worked in factories. The feedback from these workers was that the questionnaire was complex and required simple language for it to be understood. Hence the questionnaire was made simpler in language and re distributed. Reliability was assessed using Cronbach’s alpha, yielding a score of 0.80, which was judged and accepted. Three experts (Prevention of HIV/AIDS Among Migrant Workers in Thailand [PHAMIT Project] Thailand; NaKhon Phanom University, Thailand; Mahasarakham University, Thailand) inspected and commented on the draft questionnaire, then revision was made to improve its validity. It was also validated by Khon Kaen University Ethics Committee. The study used a structured questionnaire. The question was applied to the concept of access developed by Penschansky & Thomas in 1981 ^10^, which stated that access is a fit between patient need and actual outcome.

### Data analysis

The data collection process was conducted by approaching a migrant at either their home or work place. Subsequently, the migrant workers were asked to respond to a structured questionnaire interview. All participants were interviewed by trained bilingual interviewers face-to-face. After data collection, the data was validated, coded and analysed using STATA ^®^ (ver. 13; College Station, TX, USA: Stata Corp).

In part 2 of the questionnaire “Knowledge of right and benefit in health insurance of migrant workers” 0, correct; 1, wrong. In part 3 “Expectation and satisfaction from health service” and part 4 “Access to health service”, three choices were offered; however, in STATA (multiple logistic regression), there was provision only for two choices, 0 and 1. Hence the choices 1, 2, 3 had to be limited to 0 and 1: 1,high or moderate;0,low (in dataset: 1, low; 2, moderate; 3, high).

Descriptive statistics were used to examine the characteristics of migrant workers and the prevalence of access. Associations between independent variables and access to health services were calculated by using multiple logistic regression.

### Ethics statement

The researcher submitted the approval request to the Office of the Khon Kaen University Ethics Committee in Human Research, which was approved (approval number, HE 592096). A coding scheme was used for data collection, and every document relating to the participants, such as the questionnaire, was destroyed on completion of research.

Only oral consent and no written consent was obtained from all participants prior to participation. Only oral consent was obtained in order to protect the rights of the participants, since they wanted their information to be confidential (participants were worried that if they provided written consent, they would be vulnerable to government checks as they are from LAO and not citizens of Thailand).

## Results

### Characteristics of migrant workers

The characteristics of the migrant workers are shown in Table 1. The results indicated that from the total of 621 legal migrant workers, the majority of these individuals were female (63.9%), married (54.3%) with the average age of 29±8.61 years old. Most of the workers worked at restaurants (80.0%), whereas only 20.0% were in agricultural sectors. The majority had a monthly income < 9,000 Baht. About one-third of the migrant workers were ill (37.2 %) in the past year.

**Table 1.** Characteristics of migrant workers in the Northeast of Thailand (n = 621).

Characteristics	Number	Percent
**Sex**		
Male	224	36.1
Female	397	63.9
**Age (years)**		
<25	231	37.2
25–35	245	39.4
35–45	95	14.3
>45	50	8.1
Mean±SD	29.07±8.61	
Median (Min:Max)	27.0 (18:59)	
**Marital status**		
Single	284	45.7
Married	337	54.3
**Education**		
Uneducated	252	40.6
Educated	369	59.4
**Income (Baht**)		
≤9,000	447	72.1
>9,000	173	27.9
Mean±SD	6535.5±3377.4	
Median (Min:Max)	6000 (1500:25000)	
**Occupation**		
Agriculture sector	124	20.0
Employment in restaurant/factory	497	80.0
**Experience of illness**		
Not ill	390	62.8
Ill	231	37.2
**Distance (km)**		
≤5	454	73.1
>5	167	26.9
Mean±SD	4.82±4.30	
Median (Min:Max)	3 (1:25)	
**Knowledge of health insurance card**		
No	123	19.8
Yes	498	80.2
**Residency type**		
Live alone	225	36.2
Live with family/employer	396	63.8

### Accessibility to health services

Even though 37.2% of the migrant workers were ill during the past one year, only 14% (95% CI: 11–17%) of migrant workers had access to health services ( Table 2). The common illness that was found among migrant workers were musculoskeletal disorders (7.57%), diabetes mellitus (5.61%), antenatal care (4.76%), hypertension (2.21%) and allergy (1.76%). The average distance from their residence to the public hospital was 4.82±4.30 km, with 73.1% at a distance <5 km.

**Table 2.** Crude odds ratio obtained from performing bivariate analysis of each factor and accessibility to health service of migrant workers (n = 621).

Factors	Number	Access %	Crude OR	95% CI	p-value
**Overall**	621	14.0		11–17	
**Sex**					0.564
Male	224	13	1		
Female	397	14.6	1.15	0.71–1.86	
**Age (years)**					0.001
<25	231	9.1	1		
25–35	245	12.2	1.40	0.77 – 2.52	
35–45	95	42.2	3.57	1.88 – 6.77	
>45	50	42.1	2.82	1.26 – 6.31	
**Income (Baht**)					<0.001
≤9,000	447	16.3	1		
>9,000	173	8.1	0.45	0.25 – 0.81	
**Marital status**					<0.001
Single	284	8.1	1		
Married	337	19.0	2.66	1.60 – 4.41	
**Level of education**					0.526
Uneducated	252	15.1	1		
Educated	369	13.3	0.86	0.55 – 1.36	
**Occupation**					0.191
Agriculture sector	124	17.3	1		
Employment in restaurant/ factory	497	13.1	0.70	0.41 – 1.18	
**Experience of illness**					<0.001
Not ill	390	9.2	1		
Ill	231	22.1	2.79	1.75 – 4.43	
**Distance (km)**					0.528
≤5	454	14.5	1		
>5	167	12.6	0.85	1.50 – 1.43	
**Knowledge of health** **insurance card**					<0.01
No	123	21.1	1		
Yes	498	12.3	0.52	0.31 – 0.87	
**Residency type**					<0.001
Live alone	225	21.3	1		
Live with family/employer	396	9.89	0.40	0.25 – 0.64	

### Crude odds ratio obtained from performing bivariate analysis of each factor and accessibility to health services for migrant workers

Factors that had a relationship with access to health care service were age, income, marital status, occupation, the experience of illness during the past one year, knowledge of health insurance card, and place of residence, and these underwent simple logistic regression. Only the factors that had p<0.25 in the simple logistic regression were selected for further multivariate analysis using multiple logistic regression ( Table 2).

### Multivariable analysis for associated factors of accessibility to health services of migrant workers

The multivariable analysis identified only two factors that were associated with migrant workers access to health services. These factors were being married (adj. OR = 2.73; 95%CI: 1.39 – 3.90) and being ill during the past one-year (adj. OR = 2.48; 95% CI: 1.55 – 3.97). The results are shown in Table 3.

**Table 3.** Multivariable analysis for associated factors of accessibility to health service of migrant workers using multiple logistic regression (n = 621).

Factors	Number	Access %	Adjusted OR	95% CI	p-value
**Marital** **status**					<0.001
Single	284	8.1	1		
Married	337	19.0	2.73	1.39 – 3.90	
**Experience** **of illness**					<0.001
Not ill	390	9.2	1		
Ill	231	22.1	2.48	1.55 – 3.97	

Raw data gathered from the questionnaire Click here for additional data file. Copyright: © 2017 Khongthanachayopit S and Laohasiriwong W 2017 Data associated with the article are available under the terms of the Creative Commons Zero "No rights reserved" data waiver (CC0 1.0 Public domain dedication).

## Discussion

About one-third of the migrant workers who participated in the current study were ill during the past year (37.2%). However, the most common illness was musculoskeletal disorders and general illness. This may be related to the work that the migrants performed, since most of them work at restaurants, factories and in the agricultural fields. The results were similar to migrant farmworkers in the Northern Shenandoah Valley, in whom the most common health problems reported were musculoskeletal pain ^14^.

The migrant workers seldom had severe health problems, maybe because they were mostly of an age that is usually healthy. In addition, all legal migrant workers had to have a physical examination before being allowed to register with the Ministry of Labor. This study, in accordance with another study in. Thailand ^15^, stated that even though many Myanmar workers had access to the health service, around half of the migrants would not go to the health centers until their conditions worsened. This study found very poor access to health services (14%), which is a different result from a study among immigrants in Portugal, which stated that 77% of immigrants reported having used health services ^16^.

In health care utilization amongst Shenzhen migrant workers who reported illness, 62.15% did not visit a doctor because of inability to pay ^17^, which is the same reason why immigrants in Thailand in this study did not visit health services (72.1% ) - as they had a low income, less than 9,000 baht per month. Therefore, the main barriers to health access for the urban poor related to interacting effects of poverty ^18^. Migrants did not use the health service in spite of the workers having a health insurance card and the distance from home to health center was not too far. This is in contrasts to another study that found that the most common reasons for non-utilization of a medical card was a lack of transportation and lack of knowledge of where to go for care ^19^.

The multivariate analysis indicated that only two factors were associated with access to health services among migrant workers when controlling for other covariates. The first factor was that they experienced illness during the past year (adj. OR = 2.32; 95%CI: 1.40 – 3.90; p-value <0.001). Those with chronic illnesses had a high cost of health services, so the migrant workers used the service of the hospital whereas those with mild musculoskeletal disorders seldom used the health service card. They were used only for chronic illness, as treatment was expensive. In nearly all cases, poorer physical and mental health was a significant predictor of increased utilization. Perceived need and self-rated health were also associated with health services used in some studies ^20^.

The second factor was marital status (adj. OR = 2.48; 95%CI: 1.54 – 3.97; p-value <0.001): those that were married might have better support from their partners to access the health service, and migrants could share news and information about the health services within their families. Moreover, they could get more social support from others when they had health problems. According to Babitsch 2012 ^20^ which was a systematic review of studies from 1998–2011, married individuals use health services more than single individuals. In addition, Australian women who were separated, divorced, or living with children used a general practitioner more compared to their counterparts.

## Conclusion

The overall prevalence of access to health services among migrant workers was 14%, which was rather low when compared to the prevalence of illness at 37.2%. The findings support that personal factors were statistically associated with access to health service. Those who had experienced illness during the past year would seek health services to cure their health problems, especially among those with severe illness and those who received support from family.

## Data availability

The data referenced by this article are under copyright with the following copyright statement: Copyright: © 2017 Khongthanachayopit S and Laohasiriwong W

Data associated with the article are available under the terms of the Creative Commons Zero "No rights reserved" data waiver (CC0 1.0 Public domain dedication). 

Dataset 1: Raw data gathered from the questionnaire. doi, 10.5256/f1000research.11651.d165357 ^21^

## Supplementary material

Supplementary File 1: Questionnaire asked to migrants workers, relating to accessibility of health services.
