# Supplementary material for: Accessibility to health services among migrant workers in the Northeast of Thailand
Source: F1000Res. 2017 Jun 22;6:972. [Version 1] doi: 10.12688/f1000research.11651.1 (PMC5615766; doi:10.12688/f1000research.11651.1)
Supplement: Supplementary file 2 [file f1000research-6-12585-s0001.tgz › 2999c14c-f60b-4fcb-8187-e99897125d81.doc]

**Number [	][	][	]**

**Accessibility to health services and its associated factors among migrant workers in the North East of Thailand**

This questionnaires objective is to study the situation of migrant workers and identify the factors that are associated with health service among migrant workers in Northeast part of Thailand. This is a part of my dissertation,

Please answer the questions frankly in your opinion. Your answers are going to benefit me for this study. All of answers will be confidential.

This questionnaire has 3 parts which include

1 .Characteristic

2. Knowledge of right and benefit in health insurance

3. Expectation and satisfaction

4. Access to health service

As a researcher I hope to have a good cooperation from you. Thank you.

Suprawee Khongthanachaopit

Doctoral student

Faculty of public health

Khon Kaen University

**Part 1 Characteristic**

**Introduction: Kindly tick** ✓**mark the answer in** **the given column**

| **Characteristic** | | | | | | | | **Researcher** |
| --- | --- | --- | --- | --- | --- | --- | --- | --- |
| 1. | Sex | | | | | | | A1 ( ) |
|  |  | | ( ) 1. Male | | | ( ) 2. Female | |  |
| 2. | Age………………..….. years | | | | | | | A2( ) |
| 3. | Marital status | | | | | | | A3( ) |
|  |  | | ( )1Married | ( ) 2. Single | | | ( ) 3. [Dictionary](http://dictionary.sanook.com/search/ม่าย) |  |
|  |  | | ( ) 4. Divorce | ( ) 5. Separated | | |  |  |
| 4. | Level of [**education**](http://www.dek-eng.com/2389/คำศัพท์ภาษาอังกฤษ/ศัพท์ภาษาอังกฤษ-ทั่วไป/คำศัพท์ภาษาอังกฤษ-เกี่ยวกับวุฒิการศึกษา-(Education-Vocabulary).html) | | | | | | | A4 ( ) |
|  |  | | ( )1. Primary | | |  | |  |
|  |  | | ( )2. Uneducated | | |  | |  |
|  |  | | ( )3. Higher than secondary | | |  | |  |
| 5. | Income (Bath)  ( ) <3,000 ( ) 3,001-6,000  ( ) 6,001-9,000 ( ) >9,001 | | | | | | | A5 ( ) |
| 6. | Health insurance in Thailand | | | | | | | A6 ( ) |
|  |  | ( ) 1. Health insurance of migrant worker | | | | | |  |
|  |  | ( ) 2. Health insurance company | | | | | |  |
| 7. | Living status in Thailand | | | | | | | A7 ( ) |
|  |  | ( ) 1. Live with their employer | | | ( ) 2. Living alone | | |  |
|  |  | ( ) 3. Live with the family | | | ( ) 4. Live with friend | | |  |

| **Characteristic** | | | | | | | **Researcher** |
| --- | --- | --- | --- | --- | --- | --- | --- |
| 8. | Travel to Thailand | | | | | | A8 ( ) |
|  |  | | | ( ) 1. By boat  ( ) 3. By car | |  |  |
|  |  | | | ( ) 3. By bus | |  |  |
|  |  | | | ( ) 5. By train | |  |  |
| 9. | Working in Thailand | | | | | | A9 ( ) |
|  |  | | | ( ) 1. Restaurant | | ( ) 2. Agricultural sector |  |
| 10. | Working in Thailand | | | | | | A10 ( ) |
|  |  | | ( ) 1. Farmer | | | ( ) 2. Private business |  |
|  |  | | ( ) 3. House keeper | | | ( ) 4. Factory |  |
|  |  | | ( ) 5. Restaurant | | |  |  |
| 11. | Duration that stayed in Thailand  ( ) 1. <5 years ( ) 2. >5 years | | | | | | A11( ) |
| 12. | Experience of illness | | | | | | A12( ) |
|  |  | | ( ) 1. Not ill | | | |  |
|  |  | | ( ) 2. Ill | | | |  |
|  |  | |  | | ( ) 2.1 Disease | | A12.1 ( ) |
|  |  | |  | | ( ) 2.2 Muscle pain | | A12.2 ( ) |
|  |  | |  | | ( ) 2.3 Diabetes Mellitus | | A12.3 ( ) |
|  |  | |  | | ( ) 2.4 Pregnancy | | A12.4 ( ) |
|  |  | |  | | ( ) 2.5 Hypertension | | A12.5 ( ) |
|  |  | |  | | ( ) 2.6 Thyroid | | A12.6 ( ) |
|  |  | |  | | ( ) 2.7 Gout | | A12.7 ( ) |
|  |  | |  | | ( ) 2.8 Allergy | | A12.8 ( ) |
|  |  | |  | | ( ) 2.9 Pyelonephritis | | A12.9 ( ) |
|  |  | |  | | ( ) 2.10 Asthma  (…)2.11 Tuberculosis  (...) 2.12 . Others (please identify).................................................... | | A12.10 ( )  A12.11 ( )  A12.12( ) |
| 13. | Perceived severity of illness | | | | | | A13( ) |
|  |  | ( ) 1. Mild | | | | ( ) 2. Severe |  |

| **Characteristic** | | | | | | | | **Researcher** |
| --- | --- | --- | --- | --- | --- | --- | --- | --- |
| 14. | Distance from residence to health care center | | | | | | |  |
|  | 14.1 Medical Clinic | | | | | | about............kilometers | A14.1 (…...) |
|  | 14.2 Public hospital | | | | | | about.............kilometers | A14.2 (…...) |
|  | 14.3 Private hospital | | | | | | about ............ kilometers | A14.3 (…...) |
|  | 14.4 Others (please identify)....................................... | | | | | | about..............kilometers | A14.4 (……) |
|  | 14.5 Others (please identify)....................................... | | | | | | about............. kilometers | A14.5 (……) |
|  | 14.6 Others (please identify)....................................... | | | | | | about............ kilometers | A14.6 (……) |
| 15. | In the past one year  Did you ever been to hospital or not | | | | | | | A15 ( ) |
|  |  | ( ) 1. Used to | | | | | |  |
|  |  | ( ) 2. Never used to | | | | | |  |
|  |  |  | ( ) 2.1 Curative service | | | | | A15.1 ( ) |
|  |  |  |  | ( ) 1. Never | | | |  |
|  |  |  |  | ( ) 2. Used to | | | | A15.1.2 ( ) |
|  |  |  |  |  | ( ) 1. Medical clinic | | |  |
|  |  |  |  |  | ( ) 2. Public hospital | | |  |
|  |  |  |  |  | ( ) 3. Private hospital | | |  |
|  |  |  |  |  | ( ) 4. Others (please identify).......................................................... | | |  |
|  |  |  | ( ) 2.2 Health promotion | | | | | A15.2 ( ) |
|  |  |  |  | ( ) 1. Never | | | |  |
|  |  |  |  | ( ) 2. Used to | | | | A15.2.2 ( ) |
|  |  |  |  |  | ( ) 1. Medical clinic | | |  |
|  |  |  |  |  | ( ) 2. Public hospital | | |  |
|  |  |  |  |  | ( ) 3. Private hospital | | |  |
|  |  |  |  |  | ( ) 4. Others (please identify)...................................................... | | |  |
|  |  |  |  |  |  |  | |  |
|  |  |  |  |  |  |  | |  |
|  |  |  |  |  |  |  | |  |
|  |  |  |  |  |  |  | |  |

|  | **Characteristic** | | | | | **Researcher** |
| --- | --- | --- | --- | --- | --- | --- |
|  |  |  | ( ) 2.3 Prevention | | | A15.3 ( ) |
|  |  |  |  | ( ) 1. Never | |  |
|  |  |  |  | ( ) 2. Used to | | A15.3.2 ( ) |
|  |  |  |  |  | ( ) 1. Medical clinic |  |
|  |  |  |  |  | ( ) 2. Public hospital |  |
|  |  |  |  |  | ( ) 3. Private hospital |  |
|  |  |  |  |  | ( ) 4. Others (please identify).................................................... |  |
|  |  |  | ( ) 2.4 Rehabilitation | | | A15.4 ( ) |
|  |  |  |  | ( ) 1. Never | |  |
|  |  |  |  | ( ) 2. Used to | | A15.4.2 ( ) |
|  |  |  |  |  | ( ) 1. Medical clinic |  |
|  |  |  |  |  | ( ) 2. Public hospital |  |
|  |  |  |  |  | ( ) 3. Private hospital |  |
|  |  |  |  |  | ( ) 4. 4. Others (please identify).................................................... |  |

**Part 2 Knowledge of right and benefit in health insurance of migrant workers**

**Introduction: Kindly tick** ✓**mark the answer in** **the given column**

| **No** | | **Topic** | **correct** | **wrong** | **Researcher** |
| --- | --- | --- | --- | --- | --- |
| 1. | | Migrant workers have the right to own the health insurance card |  |  | B1 ( ) |
| 2. | | Children of migrant worker can buy health insurance card |  |  | B2 ( ) |
| 3. | | Health insurance card can be due as the date in back of card |  |  | B3 ( ) |
| 4. | | Health insurance card must pay 30 Baths every time that medical use |  |  | B4 ( ) |
| 5. | | If using health care in different hospital had to get refer form |  |  | B5 ( ) |
| 6. | Employer had to pay for health insurance cad every month | |  |  | B6( ) |
| 7. | Health insurance card can use only in government hospital | |  |  | B7( ) |
| 8. | Can use outpatient department after office hours | |  |  | B8( ) |
| 9. | Not confident to use the health care services without the employer’s accompanying | |  |  | B9( ) |
| 10. | Have to pay out of pocket 60 Baht for medical expense when had 2 diagnosis | |  |  | B10 ( ) |
| 11. | Have the right in using health insurance when they get sick or have an accident. | |  |  | B11( ) |
| 12. | Must have to pay for medical expense when they use a prenatal service or having vaccination and contraception | |  |  | B12( ) |
| 13. | Not eligible to use the health insurance for an automobile accident | |  |  | B13( ) |
| 14. | Eligible to use the health insurance card for tooth removal | |  |  | B14( ) |
| 15. | Not eligible to use the health insurance cards for dentures | |  |  | B15( ) |
| 16. | Not eligible to use the health insurance card for the treatment of mental diseases | |  |  | B16( ) |
| 17. | Not eligible to use the health insurance card to get the antidote HIV virus | |  |  | B17( ) |
| 18. | Eligible to use the health insurance card for the last stage renal failure treatment | |  |  | B18( ) |
| 19. | Eligible to use the health insurance card for hospital food and a private room | |  |  | B19( ) |
| 20. | Hold the health insurance card for infertility treatment | |  |  | B20( ) |
| 21 | Eligible to use the health insurance card for infertility | |  |  | B21( ) |

**Part 3 Expectation and satisfaction from health service**

**Introduction: Kindly tick mark the answer in the given column**

| **ข้อ** | **Expectation** | | | **Researcher** | **Detail** | **Satisfaction** | | | **Researcher** |
| --- | --- | --- | --- | --- | --- | --- | --- | --- | --- |
|  | **low** | **moderate** | **high** |  |  | **low** | **moderate** | **high** |  |
| **Availability** | | | | | | | | | |
| 1. |  |  |  | C1( ) | Public hospital had health service that covered all types of health service such as curative, health promotion, prevention , rehabilitation |  |  |  | D1 ( ) |
| 2. |  |  |  | C2( ) | Number of physicians and other health personnel are specialists for curative health service |  |  |  | D 2( ) |
| 3. |  |  |  | C3( ) | You could get curative health service in case of emergency and specialist other than clinic |  |  |  | D3 ( ) |
| **Accessibility** | | | | | | | | | |
| 4. |  |  |  | C4( ) | Distance from residence to public hospital are appropriate |  |  |  | D4( ) |
| 5. |  |  |  | C5( ) | Geographic or location of public health service lets you feel convenient as the distance was not too far |  |  |  | D 5( ) |
| 6. |  |  |  | C6( ) | There are vans to pick up during emergencies all the time |  |  |  | D 6( ) |

| **No** | **Expectation** | | | **Researcher** | **Detail** | **Satisfaction** | | | **Researcher** |
| --- | --- | --- | --- | --- | --- | --- | --- | --- | --- |
|  | **low** | **moderate** | **high** |  |  | **low** | **moderate** | **high** |  |
| **Accommodation** | | | | | | | | | |
| 7. |  |  |  | C7( ) | Health personnel pay attention to your wants in health service |  |  |  | D7 ( ) |
| 8. |  |  |  | C8( ) | The service are fast and convenient. Waiting time is short |  |  |  | D 8( ) |
| 9. |  |  |  | C9( ) | The guidelines of health service are easy to follow |  |  |  | D9 ( ) |
| **Affordability** | | | | | | | | | |
| 10. |  |  |  | C10( ) | They have the right to get health service because they get immigrant card |  |  |  | D10( ) |
| 11. |  |  |  | C11( ) | Due to monthly income they can pay for health service |  |  |  | D 11( ) |
| 12. |  |  |  | C12( ) | They feel comfortable to pay out of pocket money if needed |  |  |  | D12 ( ) |
| **Acceptability** | | | | | | | | | |
| 13. |  |  |  | C13( ) | The physician diagnosis the disease correctly |  |  |  | D13 ( ) |
| 14. |  |  |  | C14( ) | Hospital have medical equipment enough for curative service |  |  |  | D14 ( ) |
| 15. |  |  |  | C15( ) | They accept the given medicine |  |  |  | D15 ( ) |

**Part 4 Access to health service**

**4.1 Curative**

| **No** | **Curative** | **Access** | | | **Researcher** |
| --- | --- | --- | --- | --- | --- |
|  |  | **low** | **moderate** | **high** |  |
| 1 | Have you ever been to the OPD in hospital or not? |  |  |  | E1( ) |
| 2 | Did you ever go to the Emergency Room? |  |  |  | E2( ) |
| 3 | Were you ever admitted in a hospital? |  |  |  | E3( ) |
|  |  | | | | |
| **Availability** | | | | | |
| 4 | Did you get curative service as you wanted? |  |  |  | E4( ) |
| 5 | Did the health center have enough health personnel? |  |  |  | E5( ) |
| 6 | Did the health center have expert in curative service? |  |  |  | E6( ) |
| 7 | Did the health center have specialist clinic available? |  |  |  | E7( ) |
| **Accessibility** | | | | | |
| 8 | Was it easy to travel to health center? |  |  |  | E8( ) |
| 9 | Was the problem not being able to pay for service? |  |  |  | E9( ) |
| 10 | Was the distance too far? |  |  |  | E10( ) |
| 11 | Was an emergency van always available? |  |  |  | E11( ) |
| **Accommodation** | | | | | |
| 12 | Did you ever have service fast? |  |  |  | E12( ) |
| 13 | Did you have help in availing facilities? |  |  |  | E13( ) |

| **No** | **Curative** | **Access** | | | **Researcher** |
| --- | --- | --- | --- | --- | --- |
|  |  | **low** | **moderate** | **high** |  |
| 14 | Do you feel comfortable when received health service attention? |  |  |  | E14( ) |
| 15 | Did you have guideline to get health service? |  |  |  | E15( ) |
| **Affordability** | | | | | |
| 16 | Did you get health insurance? |  |  |  | E16( ) |
| 17 | Did you have to pay out of pocket in case of necessity? |  |  |  | E17( ) |
| 18 | Could you earn enough money to pay? |  |  |  | E18( ) |
| 19 | Did the payment are reasonable price? |  |  |  | E19( ) |
| **Acceptability** | | | | | |
| 20 | Did the health personnel give you respect? |  |  |  | E20( ) |
| 21 | Did the health personnel pay attention to service? |  |  |  | E21( ) |
| 22 | Did you have trust in health personnel? |  |  |  | E22( ) |
| 23 | Did you get health service equal to Thai people? |  |  |  | E23( ) |

**4.2 Health promotion**

| **No** | **Health promotion** | **Access** | | | **Researcher** |
| --- | --- | --- | --- | --- | --- |
|  |  | **low** | **moderate** | **high** |  |
| 1. | Did you ever got brochure or public advertisement for health promotion |  |  |  | F1( ) |
| 2. | Did you ever received health education from health personnel |  |  |  | F2( ) |

| **No** | **Health promotion** | **Access** | | | **Researcher** |
| --- | --- | --- | --- | --- | --- |
|  |  | **low** | **moderate** | **high** |  |
| 3. | Did you ever get counselling about health promotion? |  |  |  | F3 ( ) |
|  |  | | | | |
| **Availability** | | | | | |
| 4 | Did you get health promotion service as you wanted**?** |  |  |  | F4( ) |
| 5 | Did the health center have enough health personnel? |  |  |  | F5( ) |
| 6 | Did the health center have expert in health promotion service? |  |  |  | F6( ) |
| 7 | Did the health center have specialist clinic available? |  |  |  | F7( ) |
| **Accessibility** | | | | | |
| 8 | Was it easy to travel to health center? |  |  |  | F8( ) |
| 9 | Was the problem not being able to pay for service? |  |  |  | F9( ) |
| 10 | Was the distance too far? |  |  |  | F10( ) |
| 11 | Was an emergency van always available? |  |  |  | F11 ( ) |
| **Accommodation** | | | | | |
| 12 | Did you ever have service fast? |  |  |  | F12( ) |
| 13 | Did you have help in availing facilities? |  |  |  | F13( ) |
| 14 | Do you feel comfortable when received health service attention? |  |  |  | F14 ( ) |
| 15 | Did you have guideline to get health service? |  |  |  | F15( ) |
| **Affordability** | | | | | |
| 16 | Did you get health insurance? |  |  |  | F16( ) |
| 17 | Did you have to pay out of pocket in case of necessity? |  |  |  | F17( ) |
| 18 | Could you earn enough money to pay? |  |  |  | F18( ) |

| **No** | **Health promotion** | **Access** | | | **Researcher** |
| --- | --- | --- | --- | --- | --- |
|  |  | **low** | **moderate** | **high** |  |
| 19 | Did the payment are reasonable price? |  |  |  | F19 ( ) |
| **Acceptability** | | | | | |
| 20 | Did the health personnel give you respect? |  |  |  | F20( ) |
| 21 | Did the health personnel pay attention to service? |  |  |  | F21( ) |
| 22 | Did you have trust in health personnel? |  |  |  | F22( ) |
| 23 | Did you get health service equal to Thai people? |  |  |  | F23( ) |

**4.3 Prevention**

| **No** | **Prevention** | **Access** | | | **Researcher** |
| --- | --- | --- | --- | --- | --- |
|  |  | **low** | **moderate** | **high** |  |
| 1. | Did you ever get prevention service such as vaccine or condom? |  |  |  | G1( ) |
| 2. | Did you ever get knowledge about AIDS, TB, Malaria, Sexual Transmission Disease? |  |  |  | G2( ) |
| 3. | Did you ever check-up such as blood pressure monitoring, blood test? |  |  |  | G3( ) |
|  |  | | | | |
| **Availability** | | | | | |
| 4 | Did you get prevention service as you wanted? |  |  |  | G4( ) |

| **No** | **Prevention** | **การเข้าถึงบริการ** | | | **Researcher** |
| --- | --- | --- | --- | --- | --- |
|  |  | **low** | **mod** | **high** |  |
| 5 | Did the health center have enough health personnel? |  |  |  | G5( ) |
| 6 | Did the health center have expert in prevention service? |  |  |  | G6( ) |
| 7 | Did the health center have specialist clinic available? |  |  |  | G7( ) |
| **Accessibility** | | | | | |
| 8 | Was it easy to travel to health center? |  |  |  | G8( ) |
| 9 | Was the problem not being able to pay for service? |  |  |  | G9( ) |
| 10 | Was the distance too far? |  |  |  | G10( ) |
| 11 | Was an emergency van always available? |  |  |  | G11( ) |
| **Accommodation** | | | | | |
| 12 | Did you ever have service fast? |  |  |  | G12( ) |
| 13 | Did you have help in availing facilities? |  |  |  | G13( ) |
| 14 | Do you feel comfortable when received health service attention? |  |  |  | G14( ) |
| 15 | Did you have guideline to get health service? |  |  |  | G15( ) |
| **Affordability** | | | | | |
| 16 | Did you get health insurance? |  |  |  | G16( ) |
| 17 | Did you have to pay out of pocket in case of necessity? |  |  |  | G17( ) |
| 18 | Could you earn enough money to pay? |  |  |  | G18( ) |
| 19 | Did the payment are reasonable price? |  |  |  | G19( ) |
| **Acceptability** | | | | | |
| 20 | Did the health personnel give you respect? |  |  |  | G20( ) |
| 21 | Did the health personnel pay attention to service? |  |  |  | G21( ) |

| **No** | **Prevention and control** | **Access** | | | **Researcher** |
| --- | --- | --- | --- | --- | --- |
|  |  | **low** | **moderate** | **high** |  |
| 22 | Did you have trust in health personnel? |  |  |  | G22( ) |
| 23 | Did you get health service equal to Thai people? |  |  |  | G23( ) |

**4.4 Rehabilitation**

| **No** | **Rehabilitation** | **Access** | | | **Researcher** |
| --- | --- | --- | --- | --- | --- |
|  |  | **low** | **moderate** | **high** |  |
| 1. | Did you get rehabilitation facilities in case of fracture? |  |  |  | H1( ) |
| 2. | Did you rest at home after admission in hospital? |  |  |  | H2( ) |
| 3. | Did you ever get home visit or any suggestion about rehabilitation? |  |  |  | H3( ) |
|  |  | | | | |
| **Availability** | | | | | |
| 4 | Did you get rehabilitation service as you wanted? |  |  |  | H4( ) |
| 5 | Did the health center have enough health personnel? |  |  |  | H5( ) |
| 6 | Did the health center have expert in rehabilitation service? |  |  |  | H6( ) |
| 7 | Did the health center have specialist clinic available? |  |  |  | H7( ) |

| **No.** | **Rehabilitation** | **Access** | | | **Researcher** |
| --- | --- | --- | --- | --- | --- |
|  |  | **low** | **moderate** | **high** |  |
| **Accessibility** | | | | | |
| 8 | Was it easy to travel to health center? |  |  |  | H8( ) |
| 9 | Was the problem not being able to pay for service? |  |  |  | H9( ) |
| 10 | Was the distance too far? |  |  |  | H10( ) |
| 11 | Was an emergency van always available? |  |  |  | H11( ) |
| **Accommodation** | | | | | |
| 12 | Did you ever have service fast? |  |  |  | H12( ) |
| 13 | Did you have help in availing facilities? |  |  |  | H13( ) |
| 14 | Do you feel comfortable when received health service attention? |  |  |  | H14( ) |
| 15 | Did you have guideline to get health service? |  |  |  | H15( ) |
| **Affordability** | | | | | |
| 16 | Did you get health insurance? |  |  |  | H16( ) |
| 17 | Did you have to pay out of pocket in case of necessity? |  |  |  | H17( ) |
| 18 | Could you earn enough money to pay? |  |  |  | H18( ) |
| 19 | Did the payment are reasonable price? |  |  |  | H19( ) |
| **Acceptability** | | | | | |
| 20 | Did the health personnel give you respect? |  |  |  | H20( ) |
| 21 | Did the health personnel pay attention to service? |  |  |  | H21( ) |
| 22 | Did you have trust in health personnel? |  |  |  | H22( ) |
| 23 | Did you get health service equal to Thai people? |  |  |  | H23( ) |
