# Supplementary material for: Accessibility to health services among migrant workers in the Northeast of Thailand
Source: F1000Res. 2017 Jun 22;6:972. [Version 1] doi: 10.12688/f1000research.11651.1 (PMC5615766; doi:10.12688/f1000research.11651.1)
Supplement: Peer review history

# Peer review: Referee response for version 1

**DOI:** 10.5256/f1000research.12585.r23738

**Published:** 2017-09-26

**Authors:** Bhunyabhadh Chaimay

**Affiliations:** 1 Department of Public Health, Faculty of Health and Sports Science, Thaksin University, Papayom, Thailand

This is a very good article related to health services among migrant workers in Thailand, which is a hot issues. However, there are 4 issues to discuss in this article;

1. The objectives of the study mentioned in abstract, introduction and method are not relevance. Regarding the end of introduction mentioned only factors associated ….. but not mentioned about the prevalence of ….
2. Regarding the method, authors mentioned that to avoid recall bias…. In my opinion, this should be information bias.
3. About discussion, please check the accuracy of the effect size of factors marital status and experience of illness between table 3 and the discussion column 2, paragraph 3 and 4. These are not relevant to the results of the study.
4. In conclusion, the factors associated with access to health service mentioned are incomplete, which marital status factor is not mentioned yet.

I have read this submission. I believe that I have an appropriate level of expertise to confirm that it is of an acceptable scientific standard.

# Peer review: Referee response for version 1

**DOI:** 10.5256/f1000research.12585.r23741

**Published:** 2017-08-23

**Authors:** Songkramchai Leethongdee

**Affiliations:** 1 Faculty of Public Health, Mahasarakham University, Maha Sarakham, Thailand

In my view as a referee, this is a good article and suitable for publication. I would give some recommendations as follow:

1. Please review much more on migrants policy and the state direction to arrange this problem.
2. Please fill in the gap between the state policy and the situation of this problem.
3. With regards to research findings please state and recommend to policy suggestions.
4. Due to the results of his/her finding indicated that two factors were associated with access to health service among the cases, the first that they experienced illness during the past year and the second was marital status which related the previous research and evidenced. So I would suggest to author to contribute his/her own idea to respond or support the two findings as causes of problems in this article.

I do agree in this article and feel acceptable after correcting as I have recommended.

I have read this submission. I believe that I have an appropriate level of expertise to confirm that it is of an acceptable scientific standard.
